# Evaluating ChatGPT-4 in the development of family medicine residency examinations

**DOI:** 10.1371/journal.pdig.0001156

**Published:** 2025-12-19

**Authors:** Hanu Chaudhari, Christopher Meaney, Kulamakan Kulasegaram, Fok-Han Leung

**Affiliations:** 1 University Health Network, Toronto, Ontario, Canada; 2 Department of Family and Community Medicine, University of Toronto, Toronto, Ontario, Canada; 3 St. Michael’s Hospital, Unity Health, Toronto, Ontario, Canada; McGill University Faculty of Medicine and Health Sciences, CANADA

## Abstract

Creating high-quality medical examinations is challenging due to time, cost, and training requirements. This study evaluates the use of ChatGPT 4.0 (ChatGPT-4) in generating medical exam questions for postgraduate family medicine (FM) trainees. Develop a standardized method for postgraduate multiple-choice medical exam question creation using ChatGPT-4 and compare the effectiveness of large language model (LLM) generated questions to those created by human experts. Eight academic FM physicians rated multiple-choice questions (MCQs) generated by humans and ChatGPT-4 across four categories: 1) human-generated, 2) ChatGPT-4 cloned, 3) ChatGPT-4 novel, and 4) ChatGPT-4 generated questions edited by a human expert. Raters scored each question on 17 quality domains. Quality scores were compared using linear mixed effect models. ChatGPT-4 and human-generated questions were rated as high quality, addressing higher-order thinking. Human-generated questions were less likely to be perceived as artificial intelligence (AI) generated, compared to ChatGPT-4 generated questions. For several quality domains ChatGPT-4 was non-inferior (at a 10% margin), but not superior, to human-generated questions. ChatGPT-4 can create medical exam questions that are high quality, and with respect to certain quality domains, non-inferior to those developed by human experts. LLMs can assist in generating and appraising educational content, leading to potential cost and time savings.

## Author summary

In this study, we examined whether an artificial intelligence tool could assist in writing medical exam questions for family medicine residents. Producing good exam questions usually takes significant time and expertise, and many training programs struggle to keep pace with growing educational needs. We compared questions written by experienced family physicians with questions created using an AI tool and asked experts to rate their quality. We found that AI-generated questions were often similar in quality to those written by humans and frequently required advanced clinical thinking. Although experts could sometimes tell when a question was produced by AI, the overall quality remained high. These results suggest that AI could help educators create exam questions more efficiently, while still maintaining appropriate standards. We believe this approach may reduce workload and expand the availability of practice material for medical learners.

## Introduction

The development of high-quality examination questions for medical trainees is time-intensive, costly, and may not fully address all relevant educational objectives depending on the available question writers. Postgraduate resident trainees preparing for their graduating licensing examinations face challenges in the acquisition of practice material, mostly limited to institutional-based assessments, third-party educational groups, for-profit companies, or material from the licensing bodies. This presents a challenge for medical trainees in accurately identifying knowledge gaps due to concerns around the accessibility of this material. Similarly, it is difficult for postgraduate training programs to assess trainee performance longitudinally, identify trainees who need remediation, and provide them with appropriate learning resources to improve [1].

A significant development in artificial intelligence within natural language processing is Chat Generative Pre-trained Transformer (ChatGPT), a large language model (LLM) developed by OpenAI [2,3].

ChatGPT may offer faster access to feedback or information to facilitate student learning [4]. In medical education, three primary uses of artificial intelligence (AI) include learning support, assessment of student learning, and curriculum review [5,6]. There is growing interest in exploring generative AI’s capabilities to generate high-quality medical examination content [7–9].

This study aimed to characterize the quality of family medicine (FM) medical multiple-choice questions (MCQs) developed using novel AI methods (i.e., ChatGPT-4). Further, the study sought to investigate whether FM MCQ’s generated using AI methods (i.e., ChatGPT-4) were non-inferior to FM MCQs generated by human experts.

## Methods

### Study design

The primary goal of the study was to characterize the quality of FM MCQ’s generated via AI methods (i.e., ChatGPT-4); and compare the quality of AI-generated MCQ’s versus those generated by human experts.

Eight consenting attending physicians from the University of Toronto postgraduate Family Medicine training program were recruited to score questions [10]. These eight subject matter experts had previous question-writing experience on committees such as the Medical College of Canada, Royal College of Physicians and Surgeons of Canada, or the College of Family Physicians Canada licensing examinations. Recruitment specifically was targeted at physicians with a background in postgraduate family medicine question writing.

The eight physicians each scored 44 FM MCQs using the quality/scoring guide created in Table 1 across 17 quality domains (14 binary and 3 likert). Four approaches to medical question generation were considered:

**Table 1:** Question rating scales used to assess multiple choice question quality and reliability.

Question	Yes	No
Q1. Is the stem itself meaningful?		
Q2. Does the stem avoid the use of irrelevant material?		
Q3. Does the stem avoid using negatively stated language?		
Q4. Is the stem a question, as opposed to a partial sentence?		
Q5. Are the alternatives plausible?		
Q6. Are the alternatives stated clearly and concisely?		
Q7. Are the alternatives mutually exclusive?		
Q8. Are the alternatives roughly of equal length?		
Q9. Are the alternatives free from clues about which response is correct (i.e., consistent grammar, parallel form, similar in length, similar language)?		
Q10. Do the alternatives avoid the use of “all of the above” or “none of the above”?		
Q11. Are the alternatives presented in a logical order?		
Q12. Is there a reasonable number of alternatives that are plausible?		
Q13. Are complex multiple-choice items avoided (i.e., answer D is options A and B, answer E is options B and C)?		
Q14. Is the level of difficulty appropriate for the learner?		
**Question**	**Likert Scale**	
Q15. The question assesses higher order thinking (e.g., application, integration) relevant to clinical reasoning.	Strongly Disagree, Disagree, Neutral, Agree, Strongly Agree	
Q16. This question was generated using artificial intelligence.	Strongly Disagree, Disagree, Neutral, Agree, Strongly Agree	
Q17. How would you rate this multiple-choice question?	Very Poor, Poor, Fair, Good, Very Good

1. Subject matter expert generated questions/scenarios from an existing postgraduate family medicine question bank.
2. GPT-4 generated questions using cloning from existing question banks.
3. GPT-4 generated questions using novel prompts/inputs without any reference to existing questions (see Table 2).
4. GPT-4 generated questions using novel prompts/inputs (see Table 2) with human appraisal and editing.

**Table 2:** Steps and rationale for prompt engineering of novel ChatGPT generated multiple choice questions.

Step	Details/Rationale
1. Role of GPT4	Act as an educator in the setting of a postgraduate family medicine department.
2. Goal of the Task	Assist residents in preparing for their licensing examination.
3. Target Audience	Graduating family-medicine residents.
4. Objectives and Question Details	Create clinical decision-making multiple-choice questions with four options each.
5. Source Material	Base questions off the Certificant of the College of Family Physicians 105 priority topics for relevance to the jurisdiction/examination.
6. Answer and Rationale	Ensure GPT4 provides both an answer and a rationale for each question generated.
7. Level of Difficulty	Questions should be challenging enough for a graduating family medicine resident in Canada.
8. Question Stem Structure	Include the type of physician, setting of care, patient age, past medical history, medications, symptoms, physical exam findings, relevant Canadian lab values, and imaging results in the question stems.
9. Specificity in Responses	Be specific about names, doses, frequency, and duration of therapies relevant to each question.
10. Number and Focus of Questions	Instruct GPT4 to generate a specified number of questions (e.g., 10) covering defined medical topics such as diabetes, hypertension, and dyslipidemia, or provide a broader range of topics.

We utilized fourteen human-generated questions from the existing family medicine residency MCQ bank at the University of Toronto. We intended to include ten questions, however, due to human error leading to a protocol deviation, four additional questions were included, rated, and analyzed. Our a priori plan allocated an equal number of items (10/10/10/10) to each generation method to preserve balance for estimation and comparability. Owing to a protocol deviation (human error during survey set-up), 14 expert-generated items were distributed and rated. Because these items had already been scored by all raters, and removal would unnecessarily discard data and reduce precision, we retained all observations and accounted for potential imbalance with mixed-effects models. We also note this deviation as a limitation.

We created ten GPT-4 questions generated using cloning from the existing undergraduate medicine and postgraduate family medicine residency MCQ banks. Cloning involves providing GPT-4 with an existing question and asking it to create a similar question, but modifying the stem, answer, or other components of the options. We provided the question and asked GPT-4 to create similar questions based on the provided reference questions as a guide.

We created ten GPT-4 questions generated using the prompt template in Table 2.

Ten GPT-4 generated questions were created using the novel prompt template in Table 2 and were then appraised and modified by a single investigator with expertise in post-graduate medical testing (F.L.) without the use of GPT-4.

### Question rating scale

Writing an effective MCQ is a challenging task, especially in the medical context [11]. Clinical vignettes, questions, answers, and distractors must be carefully crafted to assess multiple domains of psychometric performance.

Each of the 8 subject matter experts recruited, scored each of the 44 FM MCQs with respect to 17 domains of quality. 14/17 quality measures were scored using a binary (yes/no) measurement scale; whereas, 3/17 quality measures were scored using an ordinal five-point Likert scale.

In this study, we aimed to generate a comprehensive rating scale that would address diverse quality domains (as seen in Table 1). This scale was created in collaboration with experts who have served or led institutional, provincial, and national committees for medical education (MK, FL, HC). We utilized well-validated and published MCQ rating scales [12–14] and combined these with the generally accepted best practices for evaluating MCQ items.

The eight subject matter experts scoring each FM MCQ were blinded to the generation method. Raters were not asked to answer the questions; nor were they required to provide a rationale for the quality scores they provided.

### Prompt engineering

There has been significant interest in the AI space on how to effectively generate the ideal prompt (the initial input, question, or task) for ChatGPT to maximize the efficiency and appropriateness of its response [15,16]. There have also been many plug-ins generated to accomplish this task [17]. We tested many different prompt formulations for creating an ideal medical MCQ. We present a concise 10-step, role and objective anchored prompting framework designed to elicit higher-order clinical reasoning, with explicit instructions for vignette structure, distractor quality, and jurisdiction-specific guidance (Table 2).

Table 2 summarizes the major components of the prompt that we believed were essential to be included: 1. Define the role of GPT-4 as an educator and specify the setting (i.e., postgraduate family medicine department). 2. Define the goal of the task (i.e., assist residents in preparing for licensing examination). 3. Define who the target audience/learner for the task is (graduating family-medicine resident). 4. Define the objective, question domain, type of question, and number of options (i.e., Create a clinical decision-making multiple-choice question with four options). 5. Provide a source or reference material for the questions for the unique jurisdiction/examination (i.e., based on the Certificant of the College of Family Physicians 105 priority topics). 6. Ensure GPT-4 provides an answer and rationale for the proposed question. 7. Ensure to specify the level of difficulty the question should be (i.e., challenging enough for a graduating family medicine resident in Canada). 8. Provide explicit instructions on how the stem should be structured and what type of information should be included, based on the type of examination (i.e., structure the question stems so they include the physician type, setting of care, patient age, past medical history, medications, pertinent symptoms, physical examination findings, relevant bloodwork with Canadian units for measurement, imaging results). 9. Provide instructions to be specific on names, doses, frequency, and duration of therapies as relevant to the question. 10. Instruct GPT-4 on the number of questions to generate or if it should generate questions based on a defined number of medical topics (i.e., provide 10 questions covering different topics versus 10 questions covering diabetes, hypertension, and dyslipidemia topics).

There were several reasons that this specific format was used for question generation. The common themes among other prompts were that the questions and answers generated were too simple and did not include specific patient clinical characteristics (i.e., no inclusion of age, past medical history, medications), did not identify key features relevant to the setting the physician was in, the distractors were of poor quality, the questions did not test higher-order thinking (defined as applying foundational knowledge to a case scenario to apply and integrate multiple key concepts in diagnosis and management) related to clinical decision making, and the vignettes were not sophisticated in the information provided. There were also some prompts which led to hallucinations where the answer rationale was not in keeping with appropriate clinical guidelines, however, this is an inherent limitation in the technology’s ability to source accurate and contemporary information (a challenge that family physicians also regularly face in their practice). Overall, this prompt engineering approach was chosen as the result of an iterative process testing each component to make the output as specific and detailed as possible.

### Question content

The content of questions in this study were limited to the specialty of family medicine, specifically as it is practiced in Canada, and as it would be appropriate for a graduating family medicine resident. We did not limit GPT-4 on the types of questions it could produce within family medicine. From our human-generated question bank, we randomly chose a series of questions with the only criteria being that there were no directly similar topics within the question set. Furthermore, we opted to avoid the use of any questions or stems that would rely on images, as we did not have a robust database of expert-generated questions with images to use as control variables. Given this, topics relating to dermatology, radiology, pictures of physical examination findings, and presentations involving electrocardiograms or other visual-based inputs were not included in this study.

### Hallucination quantification

A post‑hoc review of every GPT‑4–generated question was performed to identify hallucinations, specifically flagging factual errors or content that diverged from the current, high‑quality publicly available evidence. Two investigators independently reviewed every GPT-4–generated item and rationale for factual errors, outdated recommendations, or contradictions with publicly available, high-quality guidance relevant to Canadian family medicine. Discrepancies were adjudicated by a third investigator.

### Statistical analysis

Counts and percentages were used to characterize ratings of the binary quality scores. Means and standard deviations were used to characterize the Likert-scale quality scores. Likert-valued responses were dichotomized before modelling/inference.

Linear mixed effect models were fitted to each of the 17 binarized quality outcomes. The linear mixed effect models included a random effect for the rater (8 raters), a random effect for the question (44 questions), and a fixed effect for the question generation method (i.e., human expert generated, GPT-4 cloned, GPT-4 novel, GPT-4 novel with human editing). Logistic generalized linear mixed models (with an identical parameterization as described above) were considered; however, design constraints (i.e., small number of raters/questions) coupled with sparse empirical response distributions resulted in non-convergence of these seemingly more appropriate models. Hence, this study utilized a more robust/stable linear mixed probability model for inference.

Model estimated probabilities of a positive quality score, conditional on question generation method (i.e., human expert generated, GPT-4 cloned, GPT-4 novel, GPT-4 novel with human editing) were obtained for each of the 17 binarized quality outcomes. We estimated differences in conditional probabilities across question generation types, with particular interest in 3 contrasts:

1. Human expert-generated questions versus GPT-4 cloned questions.
2. Human expert-generated questions versus GPT-4 novel template questions.
3. Human expert-generated questions versus GPT-4 novel templated questions with editing.

Confidence intervals about model-estimated differences in conditional probabilities were obtained. We were interested in whether upper limits of 95% confidence intervals about differences in conditional probabilities were less than a priori stated non-inferiority margins (subjectively set at 10%) [18].

As a secondary analysis, we fit a linear mixed probability model with random rater/question effects and a fixed effect for the question generation method. In this model, the question generation method was operationalized as a binary variable (i.e., human expert generated question vs. GPT-4 generated question). Conditional probabilities of a positive quality score were estimated for each group. Differences in conditional probabilities and associated 95% confidence intervals were used to assess whether AI-generated questions were non-inferior to human-expert-generated questions, using the same 10% non-inferiority margin.

### Research ethics

Ethics approval for this study was granted from the University of Toronto Research Ethics Board (RIS #45124).

## Results

Across methods, items were rated highly for overall quality and higher-order thinking. Expert items were least likely to be perceived as AI-generated. Post-hoc review of the GPT-4 generated questions did not identify any hallucinations or inaccuracies that needed to be corrected.

Summary statistics (mean/standard deviations (SD)) for Likert valued quality domains, stratified by question generation method, are presented in Table 3. Both AI and human-expert generation methods are of high quality (Q17) and assess higher-order thinking (Q15). Human-expert-generated questions were least likely to be perceived as being AI-generated (Q16).

**Table 3:** Descriptive statistics (mean/standard deviations) for Likert valued quality domains, stratified by AI generation method.

Question	Expert (N = 112)	Cloned (N = 80)	Novel (N = 80)	Human Edited (N = 80)
**Q15. The question assesses higher order thinking (e.g., application, integration) relevant to clinical reasoning (%)**	3.90 (0.71)	3.86 (0.71)	3.75 (0.63)	3.85 (0.71)
**Q16. This question was generated using artificial intelligence (%)**	3.00 (0.91)	3.03 (0.89)	3.35 (0.86)	3.24 (0.88)
**Q17. How would you rate this multiple-choice question? (%)**	3.46 (1.00)	3.45 (0.95)	3.23 (0.83)	3.41 (1.03)

Descriptive statistics (counts/percentages) for each discretely measured quality domain, stratified by question generation method (i.e., human expert generated, GPT-4 cloned, GPT-4 novel, GPT-4 novel with human editing), are displayed in Table 4.

**Table 4:** Counts/percentages of discretely measured quality domains, stratified by question generation method (i.e., human expert generated, GPT-4 cloned, GPT-4 novel, GPT-4 novel with human editing).

Question	Response Level	Expert (N = 112)	Cloned (N = 80)	Novel (N = 80)	Human Edited (N = 80)
Q1. Is the stem itself meaningful? (%)	No	4 (3.6)	6 (7.5)	6 (7.5)	4 (5.0)
	Yes	108 (96.4)	74 (92.5)	74 (92.5)	76 (95.0)
Q2. Does the stem avoid the use of irrelevant material? (%)	No	14 (12.5)	9 (11.2)	2 (2.5)	10 (12.5)
	Yes	98 (87.5)	71 (88.8)	78 (97.5)	70 (87.5)
Q3. Does the stem avoid using negatively stated language? (%)	No	6 (5.4)	8 (10.0)	7 (8.8)	4 (5.0)
	Yes	106 (94.6)	72 (90.0)	73 (91.2)	76 (95.0)
Q4. Is the stem a question, as opposed to a partial sentence? (%)	No	5 (4.5)	0 (0.0)	0 (0.0)	2 (2.5)
	Yes	107 (95.5)	80 (100.0)	80 (100.0)	78 (97.5)
Q5. Are the alternatives plausible? (%)	No	4 (3.6)	9 (11.2)	6 (7.5)	5 (6.2)
	Yes	108 (96.4)	71 (88.8)	74 (92.5)	75 (93.8)
Q6. Are the alternatives stated clearly and concisely? (%)	No	0 (0.0)	3 (3.8)	2 (2.5)	0 (0.0)
	Yes	112 (100.0)	77 (96.2)	78 (97.5)	80 (100.0)
Q7. Are the alternatives mutually exclusive? (%)	No	45 (40.2)	27 (33.8)	36 (45.0)	43 (53.8)
	Yes	67 (59.8)	53 (66.2)	44 (55.0)	37 (46.2)
Q8. Are the alternatives roughly of equal length? (%)	No	1 (0.9)	7 (8.8)	4 (5.0)	1 (1.2)
	Yes	111 (99.1)	73 (91.2)	76 (95.0)	79 (98.8)
Q9. Are the alternatives free from clues about which response is correct (i.e., consistent grammar, parallel form, similar in length, similar language)? (%)	No	2 (1.8)	6 (7.5)	6 (7.5)	2 (2.5)
	Yes	110 (98.2)	74 (92.5)	74 (92.5)	78 (97.5)
Q10. Do the alternatives avoid the use of “all of the above” or “none of the above”? (%)	No	3 (2.7)	1 (1.2)	1 (1.2)	0 (0.0)
	Yes	109 (97.3)	79 (98.8)	79 (98.8)	80 (100.0)
Q11. Are the alternatives presented in a logical order? (%)	No	5 (4.5)	4 (5.0)	6 (7.5)	9 (11.2)
	Yes	107 (95.5)	76 (95.0)	74 (92.5)	71 (88.8)
Q12. Is there a reasonable number of alternatives that are plausible? (%)	No	9 (8.0)	7 (8.8)	9 (11.2)	6 (7.5)
	Yes	103 (92.0)	73 (91.2)	71 (88.8)	74 (92.5)
Q13. Are complex multiple choice items avoided (i.e., answer D is options A and B, answer E is options B and C)? (%)	No	2 (1.8)	0 (0.0)	16 (20.0)	0 (0.0)
	Yes	110 (98.2)	80 (100.0)	64 (80.0)	80 (100.0)
Q14. Is the level of difficulty appropriate for the learner? (%)	No	8 (7.1)	6 (7.5)	1 (1.2)	3 (3.8)
	Yes	104 (92.9)	74 (92.5)	79 (98.8)	77 (96.2)
Q15. The question assesses higher order thinking (e.g., application, integration) relevant to clinical reasoning (%)	Strongly Disagree	0 (0.0)	0 (0.0)	0 (0.0)	0 (0.0)
	Disagree	6 (5.4)	5 (6.2)	4 (5.0)	5 (6.2)
	Neutral	16 14.3)	11 (13.8)	16 (20.0)	12 (15.0)
	Agree	73 65.2)	54 (67.5)	56 (70.0)	53 (66.2)
	Strongly Agree	17 15.2)	10 (12.5)	4 (5.0)	10 (12.5)
Q16. This question was generated using artificial intelligence (%)	Strongly Disagree	1 (0.9)	1 (1.2)	0 (0.0)	1 (1.2)
	Disagree	42 (37.5)	27 (33.8)	18 (22.5)	18 (22.5)
	Neutral	26 (23.2)	21 (26.2)	18 (22.5)	24 (30.0)
	Agree	42 (37.5)	31 (38.8)	42 (52.5)	35 (43.8)
	Strongly Agree	1 (0.9)	0 (0.0)	2 (2.5)	2 (2.5)
Q17. How would you rate this multiple choice question? (%)	Very Poor	2 (1.8)	1 (1.2)	0 (0.0)	2 (2.5)
	Poor	20 (17.9)	14 (17.5)	20 (25.0)	14 (17.5)
	Fair	30 (26.8)	22 (27.5)	22 (27.5)	25 (31.2)
	Good	44 (39.3)	34 (42.5)	38 (47.5)	27 (33.8)
	Very Good	16 (14.3)	9 (11.2)	0 (0.0)	12 (15.0)

**Fig 1:**
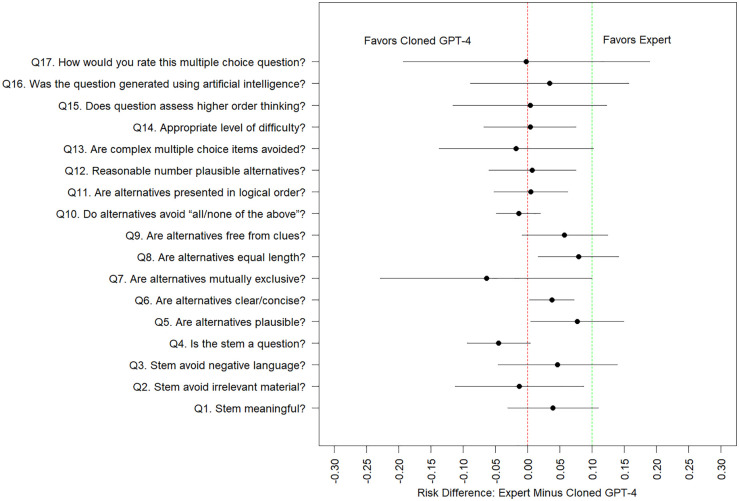
Model estimated difference in probability of a positive quality score (and associated 95% confidence interval), between questions generated by human experts versus the GPT-4 cloning method, with a superimposed 10% non-inferiority margin. *Q 16 is reverse coded and we model the probability of “disagree” or “strongly disagree”.

Model estimated probabilities of positive quality scores from Table 5 are of similar magnitude to unadjusted counts/percentages displayed in Table 4. We observe that several of the binary-valued quality scores suffer from ceiling effects (i.e., estimated probabilities near 1.0). We are particularly interested in contrasts which compare the three AI question-generated methods (GPT-4 cloned, GPT-4 novel, GPT-4 novel with human editing) versus traditional expert-generated questions as seen in Fig 1. illustrate model-estimated differences in quality scores and associated 95% confidence intervals. We superimpose a 10% non-inferiority margin and use the upper limit of the estimated 95% confidence intervals to assess whether a particular AI question generation method is non-inferior to human expert generation methods. For many of the contrasts of interest, confidence intervals are wide (because of a small number of questions/raters), and hence unanimous statements about non-inferiority cannot be made. That said, for many quality domains, and many AI question generation methods, we observe that ChatGPT-4 can generate questions which are non-inferior in quality to human experts (at a 10% non-inferiority level). Human-experts were superior to several ChatGPT-4 methods (ChatGPT-4 novel template, ChatGPT-4 novel template with human editing) at generating questions that are not perceived as AI generated (Q16).

**Table 5:** Model estimated probabilities of a positive score (and 95% confidence intervals), for each of the 17 quality domains used to evaluate family medicine multiple choice questions, stratified by question generation method (i.e., human expert generated, GPT-4 cloned, GPT-4 novel, GPT-4 novel with human editing).

Question	Human Expert	ChatGPT-4 Cloned	ChatGPT-4 Novel Template	ChatGPT-4 Novel Template with Human Editing
Q1. Is the stem itself meaningful?	0.96 (0.89, 1.00)	0.93 (0.85, 1.00)	0.93 (0.85, 1.00)	0.95 (0.87, 1.00)
Q2. Does the stem avoid the use of irrelevant material?	0.88 (0.78, 0.97)	0.89 (0.78, 0.99)	0.98 (0.87, 1.00)	0.88 (0.77, 0.98)
Q3. Does the stem avoid using negatively stated language?	0.95 (0.87, 1.00)	0.90 (0.81, 0.99)	0.91 (0.82, 1.00)	0.95 (0.86, 1.00)
Q4. Is the stem a question, as opposed to a partial sentence?	0.96 (0.92, 0.99)	1.00 (0.96, 1.00)	1.00 (0.96, 1.00)	0.98 (0.93, 1.00)
Q5. Are the alternatives plausible?	0.96 (0.89, 1.00)	0.89 (0.81, 0.96)	0.93 (0.85, 1.00)	0.94 (0.86, 1.00)
Q6. Are the alternatives stated clearly and concisely?	1.00 (0.97, 1.00)	0.96 (0.93, 0.99)	0.98 (0.94, 1.00)	1.00 (0.97, 1.00)
Q7. Are the alternatives mutually exclusive?	0.60 (0.37, 0.83)	0.66 (0.42, 0.90)	0.55 (0.31, 0.79)	0.46 (0.22, 0.70)
Q8. Are the alternatives roughly of equal length?	0.99 (0.94, 1.00)	0.91 (0.86, 0.97)	0.95 (0.89, 1.00)	0.99 (0.93, 1.00)
Q9. Are the alternatives free from clues about which response is correct (i.e., consistent grammar, parallel form, similar in length, similar language)?	0.98 (0.92, 1.00)	0.93 (0.85, 0.99)	0.93 (0.85, 0.99)	0.98 (0.90, 1.00)
Q10. Do the alternatives avoid the use of “all of the above” or “none of the above”?	0.97 (0.94, 1.00)	0.99 (0.95, 1.00)	0.99 (0.95, 1.00)	1.00 (0.97, 1.00)
Q11. Are the alternatives presented in a logical order?	0.96 (0.81, 1.00)	0.95 (0.80, 1.00)	0.93 (0.78, 1.00)	0.89 (0.74, 1.00)
Q12. Is there a reasonable number of alternatives that are plausible?	0.92 (0.76, 1.00)	0.91 (0.75, 1.00)	0.89 (0.73, 1.00)	0.93 (0.77, 1.00)
Q13. Are complex multiple choice items avoided (i.e., answer D is options A and B, answer E is options B and C)?	0.98 (0.90, 1.00)	1.00 (0.91, 1.00)	0.80 (0.71, 0.90)	1.00 (0.91, 1.00)
Q14. Is the level of difficulty appropriate for the learner?	0.93 (0.86, 1.00)	0.93 (0.85, 1.00)	0.99 (0.91, 1.00)	0.96 (0.89, 1.00)
Q15. The question assesses higher order thinking (e.g., application, integration) relevant to clinical reasoning	0.80 (0.64, 0.97)	0.80 (0.63, 0.97)	0.75 (0.58, 0.92)	0.79 (0.62, 0.95)
Q16. This question was generated using artificial intelligence ^*^	0.38 (0.20, 0.57)	0.36 (0.16, 0.54)	0.23 (0.03, 0.42)	0.24 (0.05, 0.43)
Q17. How would you rate this multiple choice question?	0.54 (0.38, 0.69)	0.54 (0.37, 0.71)	0.48 (0.30, 0.65)	0.49 (0.32, 0.66)

* Q 16 is reverse coded and we model the probability of “disagree” or “strongly disagree”.

**Fig 2:**
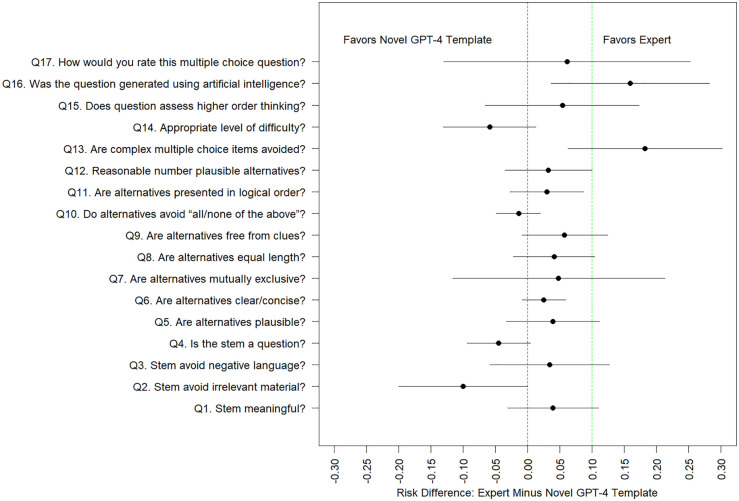
Model estimated difference in probability of a positive quality score (and associated 95% confidence interval), between questions generated by human experts versus the novel GPT-4 template method, with a superimposed 10% non-inferiority margin. *Q 16 is reverse coded and we model the probability of “disagree” or “strongly disagree”.

**Fig 3:**
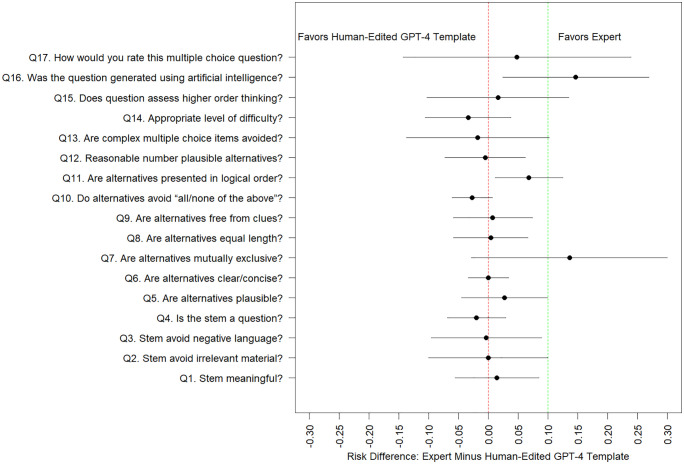
Model estimated difference in probability of a positive quality score (and associated 95% confidence interval), between questions generated by human experts versus the novel GPT-4 template method with human editing, with a superimposed 10% non-inferiority margin. *Q 16 is reverse coded and we model the probability of “disagree” or “strongly disagree”.

**Fig 4:**
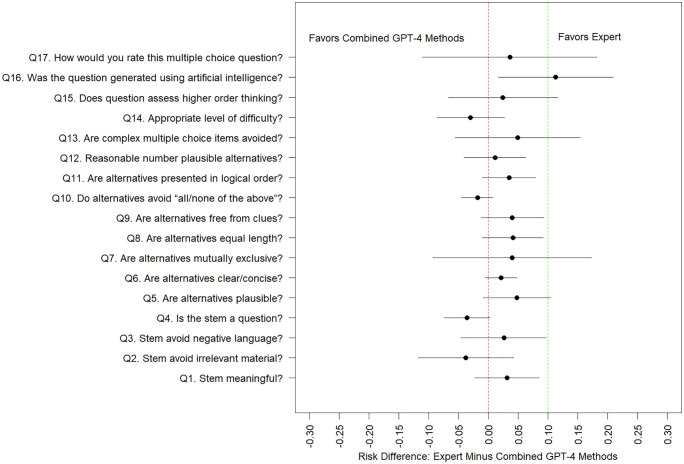
Model estimated difference in probability of a positive quality score (and associated 95% confidence interval), between questions generated by human experts versus the GPT-4 methods, with a superimposed 10% non-inferiority margin. *Q 16 is reverse coded and we model the probability of “disagree” or “strongly disagree”.

As a secondary analysis, we combined AI-generated questions (i.e., GPT-4 cloned, GPT-4 novel, GPT-4 novel with human editing) into a single group, and compared quality scores against the human expert generation method. When all AI methods are combined into a single group, we observe that for several (11/17) quality domains, AI question generation methods are non-inferior to human expert question generation methods, at a 10% non-inferiority margin (Fig 4). Human-experts were superior to combined ChatGPT-4 methods at generating questions that are not perceived as AI generated (Q16).

### Discussion

Our study found that ChatGPT-4 could generate FM MCQ’s of similar quality to human experts for several quality domains (i.e., non-inferior at a 10% margin). Human‑expert–generated questions were less likely to be identified as AI‑generated compared to ChatGPT‑4–derived questions

While the current literature suggests a possible use of AI in question generation [19,20], our study adds a rigorous evaluation of the validity of the generated questions, utilizing a novel and comprehensive MCQ rating scale [21–23]. Several diverse AI question generation methods are pragmatically compared against human expert question generation, using a novel non-inferiority design (not previously implemented when evaluating AI tools in medical education settings). While this study specifically examines a prompt engineering framework specific to GPT-4, the principles generalize to other transformer-based LLMs.

Using a novel psychometric instrument for scoring family medicine MCQ’s, our findings demonstrate that ChatGPT-4 is capable of generating MCQs with quality scores comparable to those created by human experts across a range of psychometric domains. AI’s ability to assist in generating high-quality content may significantly reduce the burden on educators while maintaining high educational standards. Our experience of question generation prior to the advent of AI suggests a writing speed of 1–2 high-quality MCQ questions per hour. AI assistance thus presents a potential benefit in efficiency and cost that must be explored. Moreover, trained expert item writers are a scarce faculty resource. Using AI assistance may enhance the capacity of newly engaged item writers and make the work of creating items less daunting for non-expert faculty.

Our structured prompting complements emerging approaches that scaffold LLM outputs for education and clinical content creation. While newer models appear less prompt-sensitive, explicit structure remains useful for ensuring item difficulty calibration, clinical specificity, and distractor plausibility.

Our results also suggest that human raters were less likely to perceive human-expert generated questions as being AI generated, as compared to several of the ChatGPT-4 methods. This reinforces the notion that AI should be viewed as a supplement to, rather than a replacement for, expert input in educational content creation. Furthermore, generative-AI use in assessment raises potential concerns about bias propagation, misinformation, and over-reliance. We recommend human-in-the-loop editorial review, transparent audit trails for item provenance, periodic guideline concordance checks, and sequestered item banks with access controls to protect exam security.

### Limitations and Future Work

One limitation to our study is that we randomized the question content, while keeping it inside the domain of family medicine. Future work is required to investigate the quality of AI generated medical MCQs developed to test knowledge in other medical sub-specialities. While the prompt engineering framework would be generalizable to these specialties, it is unclear without further research whether the intended end-users would find these questions appropriate, challenging, and factually accurate. Future work should also focus on an explicit iterative process to evaluate each component of the prompt for relevance.

Our work was limited to a small sample size of 44 MCQs with 8 raters. This was particularly limiting in the ability to make precise inferences regarding non-inferiority. As well, we did not provide training to the raters on addressing bias in AI-generated questions. Small sample sizes, coupled with ceiling effects on certain binary quality domains, also resulted in model convergence issues (hence why we used robust linear mixed probability models for inferences, rather than more standard logistic generalized linear mixed models). Future work in our group aims to address these sample size and design-related challenges in a larger cohort study.

Newer architecture with built-in chain-of-thought capabilities [24] (i.e., OpenAI o1) may reduce the need for explicit prompt decomposition, making an explicit 10-step prompt engineering less relevant. Our current work is a brief snapshot in the fast-moving field of LLM models and as they continue to evolve, further work iterating on this process is crucial. Our work may not extend to non-GPT models without further validation and our future work will aim to test our methodology with a diverse set of LLMs.

While our approach to prompting the LLM did not output any medical MCQs that were found to experience hallucinations on expert review, we did not employ any specific strategies to mitigate hallucinations. Future work should aim to employ retrieval-augmented generation [25], chain-of-verification [26], and reference checks as strategies to mitigate hallucinations and confirm the medical accuracy of the subject matter. Model updates since the time of our initial study have addressed many of these strategies and would strengthen future work aimed at producing high-quality medical questions.

As well, future work should evaluate knowledge-augmented generation (retrieval-augmented generation against current Canadian guidelines) to reduce factual drift and improve rationale quality, and test generalizability across specialties, jurisdictions, and larger item pools.

Given the novelty of this work, it is unclear how to a priori determine an appropriate non-inferiority margin (e.g., 10% versus 5%, 1% etc.). As AI evolves, equivalence or superiority designs could be argued as more appropriate, however this remains unclear at this time in the literature.

Finally, we did not have graduating family medicine residents complete the questions or evaluate them. This information would help further understand whether AI-generated questions would be an appropriate learning tool for trainees and will be the focus of our future work. We also did not explore the participants’ perspective on this process. The focus of future work should aim to understand the ethical implications, for trainees and faculty, that this poses to the current medical education curriculum.

### Conclusions

We present a novel study using GPT-4 to create medical examination questions compared to human-generated questions. We provide a framework for the prompt engineering of novel medical exam questions, a robust rating scale to evaluate the validity of AI-generated medical MCQs, and rigorously conceptualize AI versus expert comparisons in the context of a non-inferiority study design. Our study builds on the fast-growing body of work in evaluating how AI can augment medical education and serves to guide further developments for educators. Incorporating AI in question generation presents a cost-effective alternative, reducing time and resource burdens on faculty while maintaining high-quality standards.

## Supporting information

S1 File Sample GPT-4 question-generation transcript. Example prompt and output showing AI-generated family medicine exam question with answer and rationale.
(DOCX)
