# Supplementary material for: Evaluating ChatGPT-4 in the development of family medicine residency examinations
Source: PLOS Digit Health. 2025 Dec 19;4(12):e0001156. doi: 10.1371/journal.pdig.0001156 (PMC12716725; doi:10.1371/journal.pdig.0001156)
Supplement: S1 File — Example prompt and output showing AI-generated family medicine exam question with answer and rationale. (DOCX) [file pdig.0001156.s001.docx]

**Supplementary Data**

**S1 File.** Sample Novel GPT-4 Transcript

**Input:** You are to play the role of an educator in the postgraduate family medicine department. Your responsibility is to assist residents in preparing for their licensing examination following a two-year family medicine residency program. Your objective is to create a clinical decision-making multiple-choice question with four options that effectively aids residents in their exam preparation based off the CCFP 105 priority topics. Please also include an answer and the rationale for it and make them challenging enough for the level of a graduating family medicine resident in Canada. Structure the question stems so that they include the physician type, setting of care, patient age, past medical history, medications, pertinent symptoms and physical examination findings, and relevant bloodwork (with Canadian units for measurements) or imaging results, and the question. Be specific with the name, dose, frequency, and duration of therapy in the options, as relevant to the question. Provide 10 questions covering different topics.

**Output:** Creating multiple-choice questions (MCQs) that align with the CCFP 105 priority topics requires careful consideration of clinical relevance, practical application, and the integration of critical thinking skills. Here are 10 MCQs designed to aid family medicine residents in Canada in preparing for their licensing examination. These questions span various topics, challenging residents to apply their knowledge and reasoning skills in clinical scenarios.

Question 1: Diabetes Management

**Stem:** You are a family physician in a community clinic reviewing a 58-year-old male patient with a 5-year history of type 2 diabetes mellitus, currently managed with Metformin 1000 mg twice daily. His recent HbA1c is 8.2% despite dietary modifications and regular exercise. His BMI is 30 kg/m^2, and blood pressure is 132/88 mmHg. He has no history of cardiovascular disease.

**Question:** What is the next best step in managing this patient's diabetes?

**Options:**

A. Add Insulin Glargine, 10 units at bedtime.

B. Add Sitagliptin, 100 mg once daily.

C. Increase Metformin to 2000 mg twice daily.

D. Add Empagliflozin, 10 mg once daily.

**Answer:** D. Add Empagliflozin, 10 mg once daily.

**Rationale:** According to current guidelines, adding an SGLT2 inhibitor, such as Empagliflozin, is preferred in type 2 diabetes patients with cardiovascular disease risk factors due to its benefits in reducing heart failure and cardiovascular mortality, besides glycemic control. The patient's HbA1c level indicates the need for additional pharmacotherapy, and his BMI suggests that the weight loss benefit of SGLT2 inhibitors could be advantageous. Metformin is already at or near the maximum effective dose, and further increasing it may not provide significant additional glycemic control and could increase side effects. Insulin and Sitagliptin are options but do not provide cardiovascular benefits for this patient without established cardiovascular disease.
